# Supplementary material for: The impact of cell size on morphogen gradient precision
Source: Development. 2023 May 30;150(10):dev201702. doi: 10.1242/dev.201702 (PMC10281552; doi:10.1242/dev.201702)
Supplement: Supplementary file 1 [file develop-150-201702_review_history.pdf]

Impact of cell size on morphogen gradient precision

Jan A. Adelmann, Roman Vetter and Dagmar Iber

DOI: 10.1242/dev.201702

Editor: Paul Francois

Review timeline

Submission to Review Commons:	11 October 2022
Submission to Development:	13 February 2023
Editorial decision:	27 March 2023
First revision received:	27 April 2023
Accepted:	2 May 2023

Review Commons reviews

Reviewer 1

Evidence, reproducibility and clarity

Summary:

- In developing systems, morphogens gradients pattern tissues such that cells along the patterning length sense varying levels of the morphogen. This process has a low positional error even in the presence of biological noise in numerous tissues including the early embryo of the Drosophila melanogaster. The authors of this manuscript developed a mathematical model to test the effect of noise and mean cell diameter on gradient variability and the positional error they convey.

- They solved the 1D reaction-diffusion equation for N cells with diameters and kinetic parameters sampled from a physiologically relevant mean and coefficient of variation (CV). They fit the resulting morphogen gradients to a hyperbolic cosine profile and determined the decay length (DL) and amplitude (A) for a thousand independent runs and reported the CV in DL and A.

- The authors found that CV in DL and A increases with increase in mean cell diameter. They propose a mathematical relationship between CV in DL scales as an inverse-square-root of N. Whereas the CV in DL and A is a weak function of CV of cell surface area (CVa) if CVa <
1.

- They further looked at the shift in readout boundaries and compared four different readout metrics: spatial averaging, centroid readout, random readout and readout along the length of the cilium. Their results show that spatial averaging and centroid have a high readout precision.

- They finally showed that the positional error (PE) increases along the patterning length of the tissue and increases with increasing mean cell diameter.

- The authors also supported their theoretical and simulated results by looking at mean cell areas reported for in patterning tissues in literature which also have a higher readout precision with smaller cell diameters.

Major comments:

- Most of the key conclusions are convincing. However, there are four major points that should be addressed. First, the authors conclude the section titled, "The positional error scales with the

© 2023. Published by The Company of Biologists under the terms of the Creative Commons Attribution License (https://creativecommons.org/licenses/by/4.0/).

square root of the average cell diameter," by saying that morphogen systems with small cells can have high precision in absolute length scales, but not on the scale of one cell diameter. They state this would result in salt and pepper patterns in the transition zones. The authors should either support this with biological examples or explain why this is not observed experimentally.

- Second, perhaps the main conclusion of the paper is that morphogen gradients pattern best when the average cell diameter is small. The authors support this by reviewing the apical cell area of epithelial systems that are known to be patterned by morphogens and those that are not (presumably taking apical cell area as a proxy for cell diameter). However, the key parameter is not absolute cell diameter, but the cell diameter relative to the morphogen length scale. The authors should report the ratio of these two quantities in their literature analysis.

- Third, as part of their literature analysis, the authors state that in the Drosophila syncytium, there are morphogen gradients, but they imply that because these gradients operate prior to cellularization, one cannot use the large distances between nuclei as counter evidence to their main conclusion. Rather than simply dismissing the case of the Drosophila syncytium, the authors should explain why this case does not apply, using reasoning based on their model assumptions.

- Fourth, related to the above: the authors then state that there are no morphogen gradients known during cellularization. Unless I am misunderstanding their point, this is untrue. The Dpp gradient acts during the process of cellularization and specifies at least three distinct spatial domains of gene expression. Furthermore, not long after gastrulation, EGFR signaling patterns the ventral ectoderm into at least two distinct domains of gene expression. What are the cell areas in that case?

Minor comments:

- Figs 1cd:

The way the system is set-up: (DL = 20 micron, Patterning Length (LP) = 250 micron, Nominal cell diameter (D) = 5 micron) the DL/L ~ 0.08 which makes the exponential profile far to a small value around 100 micron. This means in all these simulations, the LP was only around 100 micron, cells beyond that saw nearly zero concentration.
Because of this, when diameters were varied from 0.2 - 40 micron, there could be as few as 2.5 cells in the "patterning region" which could be responsible for higher variability in DL and A.

Would any of the results change if DL/L was higher, around 0.2?

The source region is 25 microns in length and all cell diameters above 25 micron get defaulted back to 25 micron which explains the flatness lines in the region beyond mu_delta/mu_DL> 1

Results:

Pg 2 (bottom left): In the git repository code, the morphogen gradients are fit to a hyperbolic cosines function (described in reference 19) which is not described in the main text. Having this in the main text would help readers understand why fig 1c has variation in d only, D only and all k parameters whereas fig 1d has variation with all individual parameters p, d and D and all k.

- Figure 3b:

In figures where markers are overlapping perhaps the authors can use a "dot" to identify one set of simulations and a "o" to identify the ones under it. The way the plots are set up currently makes it hard for the reader to understand where certain points on the plot are.

Methods:

The Methods can be more descriptive to include certain aspects of the simulations such as adjusted lambda which is only described in the code and not the main text or supplementary.

© 2023. Published by The Company of Biologists under the terms of the Creative Commons Attribution License (https://creativecommons.org/licenses/by/4.0/).

Git code:

The git code function handles do not represent figure numbers and should be updated to make it easier for readers to find the right code

Significance

This manuscript contributes certain key aspects to the patterning domain. The three most important contributions of this work to the current literature are: (1) the scaling relationships developed here are important, (2) the idea that PE increases at the tail-end of the morphogen profile is nicely shown and (3) Comparison of various readout strategies.

Reviewer 2

Evidence, reproducibility and clarity

Summary:

How morphogen gradients yield to precise patterning outputs is an important problem in developmental biology. In this manuscript, Adelmann et al. study the impact of cell size in the precision of morphogen gradients and use a theoretical framework to show that positional error is proportional to the square root of cell diameter, suggesting that the smaller the cells in a patterning field, the more precise patterns can be established against morphogen gradient variability. This result remains true even when cells average the morphogen signal across their surface or spatial correlations between cells are introduced. Thus, the authors suggest that epithelial tissues patterned by morphogen gradients buffer morphogen variability by reducing apical cell areas and support their hypothesis by examining several experimental examples of gradient-based vs. non-gradient-based patterning systems.

Major comments:

1. While the idea that smaller cells yield to more precise morphogen gradient outputs is attractive, it is unclear whether patterning systems use this strategy to make patterns more precise, as there are several mechanisms that could achieve precision. Do actual developmental systems use it as a mechanism to increase precision? Or precision is achieved through other mechanisms (for example, cell sorting as in the zebrafish neural tube; Xiong et al. Cell, 2013). Indeed, classical patterning work on Drosophila embryo suggest that segmentation patterns are of an absolute size rather by an absolute number of cells (Sullivan, Nature, 1987). According to the authors, the patterning stripes should be more precise when embryos have higher cell densities than in the wild-type, but stripes are remarkably precise in wild-type embryos. This is likely due to other precision-ensuring mechanisms (such as downstream transcriptional repressors, in this case).

2. Their modeling approach is based on exponential gradients formed by diffusion and linear degradation, but in reality, actual morphogen gradients are affected by receptor and proteoglycan binding and are likely not simply exponential and/or interpreted at the steady state. Do the main results of the manuscript hold even for non-exponential gradients or before they reach a steady state?

3. In their Discussion section, the authors note that several patterning systems, such as the Drosophila wing and eye discs, show smaller cells near the morphogen source relative to other regions in the tissue. This observation suggests a prediction of the authors' hypothesis that can be tested experimentally. In the Drosophila wing and eye discs genetic mosaics of ectopic morphogen sources (such as Dpp) can (and have) been made. Therefore, one could predict that the patterning outputs in a region of larger cross-sectional areas will be more imprecise than in the endogenous source. Since this is a theoretical paper, it is understandable that authors are not going to make this experiment themselves, but I wonder if they can use published data to test this prediction or at least mention it in the manuscript to offer the experimental biology reader an idea of how their hypothesis can be tested experimentally.

© 2023. Published by The Company of Biologists under the terms of the Creative Commons Attribution License (https://creativecommons.org/licenses/by/4.0/).

Other comments:

- The Methods section should be expanded and should include more details about how authors consider cell size in their simulations. As presented, I believe that experimental biologists will not be able to grasp how the analysis was done.

- Authors use adjectives such as 'little' as 'small' without a comparative reference. For example in the abstract, the authors say that apical areas "are indeed small in developmental tissues." What does "small" mean? This should be avoided throughout the text.

Significance

Overall, I believe that the manuscript is well written and deserves consideration for publication. However, authors should consider the points outlined above in order to make their manuscript more accessible and relevant to the developmental biology community.

Reviewer 3

Evidence, reproducibility and clarity

In their mansucript "Impact of cell size on morphogen gradient precision" the authors Adelmann, Vetter and Iber numerically analyse a one-dimensional PDE-based model of morphogen gradient formation in tissues in which the cell sizes and cell-specific parameters locally affecting the gradient properties are varied according to predefined distributions. They find that the average cell size has the largest impact on the variance of the gradient shape and the read-out precision downstream, while other factors such as details of the readout mechanism have markedly less influence on these properties. In addition they demonstrate that averaging gradient concentrations over typical cell areas induces a shift of the readout position, which however appears to be insignificant (~1% of the cell diameter) for typical parameters.

Overall this manuscript is in very good shape already and tackles an interesting topic. I still would like the authors to address the comments below before I would recommend any publication. My main criticism pertains to some of the authors' derivations which, as I find, partly do deserve more detail, and to their conclusions about gradient readout precision.

MAJOR COMMENTS

1 - p. 1, left column: The positional error of the readout position does not only depend on the variation of the gradient parameters, as suggested by the first part of the introduction. A very important factor is also the fluctuations due to random arrival of molecules to the promoters that perform the readout due to the limited (and typically low) molecule number. In fact, for positions very distant to the source of the gradient, this noise source is expected to be dominant over gradient shape fluctuations. Importantly, these fluctuations also arise for non- fluctuating, "perfect" gradient inputs if copy numbers of the morphogen molecules are limited (which they always are). This important contribution to the noise is neglected in the work of the authors. This is OK if the purpose is focusing on the origin and influence of the gradient shape fluctuations, but that focus should be clearly highlighted in the introduction, saying explicitly that noise due to diffusive arrival of transcription factors is not taken into account in the given work (see, e.g., Tkacik, Gregor, Bialek, PLoS ONE 3, 2008)

2 - p.1, right column: Why exactly are the parameters p, d, D assumed to follow a log-normal distribution? Such distribution has been verified for cell size, but the rationale behind choosing it also for the named parameters should be explained, in particular for D. Why would D depend on local properties of the cell? Which diffusion / transport mechanism precisely is assumed here?

Moreover, is there any relationship between A_i and p_i, d_i and D_i, or are these parameters varied completely independently? If yes, is there a justification for that?

© 2023. Published by The Company of Biologists under the terms of the Creative Commons Attribution License (https://creativecommons.org/licenses/by/4.0/).

3 - p. 2, right column, section on "Spatial averaging": First of all, how is "averaging" exactly defined here? Do the authors assume that the cells can perfectly integrate over their surface in the dimensions perpendicular to their height? If yes, then this should be briefly mentioned here. Secondly, the shift \Delta x calculated by the authors ultimately seems to trace back to the fact that the cells average over an _exponential_ gradient, whose derivative also is exponential, such that levels further to the anterior from the cell center are higher (on average) than levels to the posterior of it. I suppose, therefore, that a similar calculation for linear gradients would not lead to any shift. If these things are true they deserve being mentioned in this part of the manuscript because they provide an intuitive explanation for the shift. Thirdly, in Fig. 2A the cell sizes seem exaggerated with respect to the gradient length. This seems fine for illustrative purposes, but if it is the case it should be mentioned. Also, I believe that this figure panel would benefit from showing another readout case where the average concentration e.g. in cell 1 maps to its corresponding readout position, in order to show that this process repeats in every cell. Moreover, it could be indicated that in the shown case C_\theta matches the average concentration in cell 2 at the indicated position.

As for the significance of the magnitude of the shift for typical parameters as calculated by the authors: I believe that it could be said more explicitly and clearly that under biological conditions the calculated shift overall seems insignificant, as it amounts to a small fraction of the cell diameter.

Finally, and most importantly: The term "spatial averaging" can have a different meaning in developmental biology than the one employed by the authors. While the authors mean by it that individual cells average the gradient concentration over their area, in other works "spatial averaging" typically means that individual cells sense "their" gradient value (by whatever mechanism) and then exchange molecules activated by it, which encode the read- out gradient value downstream, _between_ neighboring cells, in order to average out the gradient values "measured" under noisy conditions. The noise reduction effect of such spatial averaging can be very significant, as evidenced by this (incomplete) list of works which the authors can refer to:

- Erdmann, Howard, ten Wolde, PRL 103, 2009
- Sokolowski & Tkacik, PRE 91, 2015
- Ellison et al., PNAS 113, 2016
- Mugler, Levchenko, Nemenman, PNAS 113, 2016

The main point, however, is that this is a different mechanism as the one described by the authors, and this should be clearly mentioned in order to distinguished them. I would therefore also advise the authors to make the section title more precise here, by changing "Spatial averaging barely affects ..." to "Spatial averaging across the cell area barely affects
..." for clarity.

4 - p. 3, Figure 3: It would be good to highlight the fact that the colours in panel A correspond to the bullet colors in the other panels also in the main text.

As to the comparison of different readout strategies: How exactly were the different readout mechanisms compared on the mathematical side? More precisely: How was the readout by the whole area matched (in terms of fluxes) to the readout at a single point, be it in the center of the cell or a ranomly chosen point? How was it ensured that the comparison is done at equal footing?

p. 3, right column: "... similar gradient variabilities, and thus readout precision": Linking to comment 1 above, this is strictly speaking only the case when the only source of fluctuations in the readout is the gradient fluctuations. I would therefore leave this statement out.

5 - p. 3, section on positional error (right column): In this part I had most troubles following the thoughts of the authors.

First of all, the measure that the authors use for the positional error is sigma_x / mu_lambda, i.e. the standard deviation of the readout position relative to the gradient length. The question is

© 2023. Published by The Company of Biologists under the terms of the Creative Commons Attribution License (https://creativecommons.org/licenses/by/4.0/).

whether this is the correct measure. It should be specified what the motivation for normalizing by mu_lambda is. In the end, one could argue, what the cells really do care
about would be that the developmental process can assign cell fates with single cell precision, for which the other measure shown in Eq. (6) is the representative one. Now in contrast to the former measure, the latter actually increases with decreasing cell diameter.

Secondly, even when the former measure (sigma_x / mu_lambda) is employed, Fig. 3(D) shows that while it decreases with decreasing cell diameters, in the regime of small diameters the std. dev. of the readout position becomes larger than the average cell diameter, which actually would mean that cell fates cannot be assigned with single-cell precision. While the authors later report both quantities for specific gradients, it should be clarified beforehand which of the measures is the relevant one.

Moreover, in the following derivations, mu_x is not properly introduced. What exactly is the definition of that quantity? Is it the mean readout position? If yes, it is not clear why exactly it would be interesting and relevant to the cell. This should be properly explained in a way that does not require the reader to look up further details in another publication.

At the end of this section the authors come back to the sigma_x / mu_delta measure again and indeed point out that it increases with decreasing mu_delta, which causes a bit of confusion because the initial part of the section only talks about the increase of the pos. error with mu_delta. Overall I find that this section should be rewritten more clearly. Right now it leaves the reader with the "take home message" that small cells are good because they lead to smaller pos. error, but when the--in my opinion--relevant measure (sigma_x/mu_delta) is employed the opposite is the case. This is confusing and unclear about the authors'
intentions in that part.

Finally, the authors could also supplement the numbers that they name for the FGF8 and SHH gradients by the known numbers for the Bcd gradient in Drosophila, which has been studied excessively and constitutes a paradigm of developmental biology. Here mu_delta ~=
6.5 um, while mu_lambda ~= 100 um, such that mu_delta/mu_lambda < 1/15, which defines yet another regime than the other two gradients. It would be interesting to compare their respective numbers altogether, and also discuss the ones for Drosophila in view of the fact that in experiments sigma_x ~= mu_delta for this species.

6 - p. 4, section on the effect of spatial correlation: Here the authors chose to order the kinetic parameters in ascending or descending order. Is there any biological motivation for that particular choice? Other types of correlations seem possible, e.g. imposing the rule that successive parameter values are sampled starting from the previous value, p_i+1 = o_i +- delta_i+1 where delta_i+1 are random numbers with a defined variance.

7 - p.5, Discussion: "..., but with nuclei much wider than the average cell diameter". To be honest, I could not completely imagine what is meant with this sentence. Intuitively, it seems that the nuclei cannot be larger than the cells, but I suppose that some kind of special anisotropy is considered here? In any case, this should be made precise.

Moreover, I find that the conclusion that morphogen gradients "provide precise positional information even far away from the morphogen source" goes to far based on the authors' work, precisely for the fact input fluctuations due to limited morphogen copy number, which can become detrimentally low far away from the source, are not considered, neither the timescales needed to both establish and sample such low concentrations far away from the source. While thus, according to the work of the authors, the fluctuations in the morphogen signal may be favorably small, these other factors are supposed to exert a strong limit on positional information. This conclusion therefore seems unjustified and should be toned down, or even better taken out and replaced by a more accurate one, which only focuses on the gradient shape fluctuations, not on the conveyed positional information.

MINOR COMMENTS

- p. 1: "and find that positional accuracy is _the_ higher, the narrower the cells".

© 2023. Published by The Company of Biologists under the terms of the Creative Commons Attribution License (https://creativecommons.org/licenses/by/4.0/).

(This sentence, however, should be anyhow revised in view of major comment 5 above.)

- p. 4: "... with an even slightly smaller prefactor."

Significance

I believe that this work is significant to the community working on the theoretical foundations of morphogen gradient precision in developmental systems. The main interesting findings are that small cell diameters lead to smaller positional error (although the relevant measure should be clarified according to my comment no. 5), and that the gradient shape fluctuations are surprisingly robust with respect to the readout mechanism.

Its limitations consist of the fact that the impact of small copy numbers on the readout and associated timescales are neglected, such that the findings of the authors on gradient robustness cannot be simply transferred by simple conversion formulas to readout robustness / positional information. Comment 5 goes hand in hand with this, as a different conclusion may emerge depending on how the relevant positional error measure is defined. This should be fixed by the authors as indicated in the main part of the report.

Author response to reviewers' comments

Reviewer #1 (Evidence, reproducibility and clarity (Required)):

Summary:

In developing systems, morphogens gradients pattern tissues such that cells along the patterning length sense varying levels of the morphogen. This process has a low positional error even in the presence of biological noise in numerous tissues including the early embryo of the Drosophila melanogaster. The authors of this manuscript developed a mathematical model to test the effect of noise and mean cell diameter on gradient variability and the positional error they convey.

They solved the 1D reaction-diffusion equation for N cells with diameters and kinetic parameters sampled from a physiologically relevant mean and coefficient of variation (CV). They fit the resulting morphogen gradients to a hyperbolic cosine profile and determined the decay length (DL) and amplitude (A) for a thousand independent runs and reported the CV in DL and A.

The authors found that CV in DL and A increases with increase in mean cell diameter. They propose a mathematical relationship between CV in DL scales as an inverse-square-root of N. Whereas the CV in DL and A is a weak function of CV of cell surface area (CVa) if CVa < 1.

They further looked at the shift in readout boundaries and compared four different readout metrics: spatial averaging, centroid readout, random readout and readout along the length of the cilium. Their results show that spatial averaging and centroid have a high readout precision.

They finally showed that the positional error (PE) increases along the patterning length of the tissue and increases with increasing mean cell diameter.

The authors also supported their theoretical and simulated results by looking at mean cell areas reported for in patterning tissues in literature which also have a higher readout precision with smaller cell diameters.

© 2023. Published by The Company of Biologists under the terms of the Creative Commons Attribution License (https://creativecommons.org/licenses/by/4.0/).

Major comments:

Most of the key conclusions are convincing. However, there are four major points that should be addressed. First, the authors conclude the section titled, "The positional error scales with the square root of the average cell diameter," by saying that morphogen systems with small cells can have high precision in absolute length scales, but not on the scale of one cell diameter. They state this would result in salt and pepper patterns in the transition zones. The authors should either support this with biological examples or explain why this is not observed experimentally.

We thank the referee for pointing out this imprecise comment, which we have removed. The exact nature of transition zones between patterning domains is a subject of ongoing research in our group, and goes beyond the scope of the present work. We will be sharing our results on this aspect in a separate forthcoming publication.

Second, perhaps the main conclusion of the paper is that morphogen gradients pattern best when the average cell diameter is small. The authors support this by reviewing the apical cell area of epithelial systems that are known to be patterned by morphogens and those that are not (presumably taking apical cell area as a proxy for cell diameter). However, the key parameter is not absolute cell diameter, but the cell diameter relative to the morphogen length scale. The authors should report the ratio of these two quantities in their literature analysis.

Since cell areas and cell diameters are monotonically increasing functions of one another for reasonably regular cell shapes, we indeed consider apical cell areas as proxies for the cell diameter, as the referee correctly noted. Cell areas are more frequently reported in the literature than cell diameters, which is why we compiled these in our analysis.

We have now revised our analysis of the effect of the cell diameter on patterning precision to further length scales relevant in the patterning process. We show by example of the *Drosophila* wing disc how the parallel changes in cell diameter and morphogen source size compensate for the increase in gradient length and domain size, which would otherwise reduce patterning precision over time as the readout positions shift away from the source to maintain the same relative position in the growing wing disc.

Lamentably, accurate measurements of morphogen gradients in epithelial tissues are still rare. In fact, among the listed tissues that are patterned by gradients, we are only aware of measurements of the SHH and BMP gradients in the mouse NT (lambda = 20 μm) and of the Dpp gradients in the *Drosophila* wing and eye discs [Wartlick, et al., Science, 2011 & Wartlick et al., Development, 2014]. We agree that it would be great if experimental groups would measure this in more tissues. In this revised and extended analysis, we show that the positional error increases with the cell diameter in absolute terms, not only relative to any reference length, be it the gradient length or cell diameter.

Third, as part of their literature analysis, the authors state that in the Drosophila syncytium, there are morphogen gradients, but they imply that because these gradients operate prior to cellularization, one cannot use the large distances between nuclei as counter evidence to their main conclusion. Rather than simply dismissing the case of the Drosophila syncytium, the authors should explain why this case does not apply, using reasoning based on their model assumptions.

Our paper is concerned with patterning of epithelia (which we now make clearer in the manuscript), and we would not want to stretch our paper to other tissue types, as the reaction-diffusion process in them differs. But we do not share the referee's sentiment that the syncytium would present a counter-example. Since our model explicitly represents kinetic variability between spatial regions bounded by cell membranes, which are absent in the syncytium, our model is not directly applicable to it. We now provide this argument in the discussion, as requested by the referee.

© 2023. Published by The Company of Biologists under the terms of the Creative Commons Attribution License (https://creativecommons.org/licenses/by/4.0/).

At 100 µm [Gregor et al., Cell, 2007], the Bicoid gradient is 5 times longer than the SHH/BMP gradients in the mouse neural tube and more than 10 times the reported length of the WNT gradient in the *Drosophila* wing disc [Kicheva et al., Science, 2007]. The nuclei become smaller as they divide because the anterior-posterior length of the *Drosophila* embryo remains about 500 µm [Gregor et al., Cell, 2007], but even at the earliest patterning stage their diameter will not be larger than 10 µm at midinterphase 12 [Gregor et al., Cell, 2007, Fig. 3A].

Fourth, related to the above: the authors then state that there are no morphogen gradients known during cellularization. Unless I am misunderstanding their point, this is untrue. The Dpp gradient acts during the process of cellularization and specifies at least three distinct spatial domains of gene expression. Furthermore, not long after gastrulation, EGFR signaling patterns the ventral ectoderm into at least two distinct domains of gene expression. What are the cell areas in that case?

Unfortunately, the referee does not provide literature references, and we were not able to find anything in the literature ourselves. We have now rephrased the statement to “we are not aware of morphogen gradient readout during cellularisation”.

Minor comments: Figs 1cd:

The way the system is set-up: (DL = 20 micron, Patterning Length (LP) = 250 micron, Nominal cell diameter (D) = 5 micron) the DL/L ~ 0.08 which makes the exponential profile far to a small value around 100 micron. This means in all these simulations, the LP was only around 100 micron, cells beyond that saw nearly zero concentration. Because of this, when diameters were varied from 0.2 - 40 micron, there could be as few as 2.5 cells in the "patterning region" which could be responsible for higher variability in DL and A.

Patterning in the neural tube works across several 100 µm. At x=100µm, there is still exp(-5)=0.0067 of the signal left, which likely well translates into appreciable numbers of the morphogen molecule (see [Vetter & Iber, 2022] for a discussion of concentration ranges cells might sense). Unfortunately, very little is known about absolute morphogen numbers in the different patterning systems – experimental data is available only on relative scales, not in absolute numbers. While more quantitative experiments are still outstanding, modeling work needs to be based on reasonable assumptions. The seemingly quick decay of exponential profiles (when plotted on a linear scale) can be deceiving. In fact, exponential profiles describe the same fold-change over repeated equal distances, which makes them biologically very useful for different readout mechanisms operating on different levels of morphogen abundance. Our simulations are not limited to a patterning length of 100µm. Our work merely shows that variable exponential gradients stay precise over a long distance. We draw no conclusion on whether cells are able to interpret the low morphogen concentrations that arise far in the patterning domain - this aspect certainly deserves further research.

The referee's observation is correct in that for a cell diameter of up to 40 µm, there are only few cells in the patterning domain (namely down to about six, for a length of 250µm, as used in the simulations). It is also correct that this is the reason why gradients in such a tissue have greater variability in lambda and C0. This is precisely the main point we are making in this study: The narrower the cells in a tissue of given size, the less variable the morphogen gradients, and the more accurate the positional information they carry. Conversely, the wider the cells in x direction, the more variable the gradients.

Would any of the results change if DL/L was higher, around 0.2?

As we consider steady state gradients, nothing changes if we fix the (mean) gradient decay length and only shorten the patterning domain, except for a small boundary effect at the far end of the tissue due to zero-flux conditions applied there. At a fixed gradient length, the steady-state gradients just extend further if DL/L is increased (for example to 0.2), reaching lower concentrations, but the shape remains unchanged, and so does the morphogen concentration at a given absolute readout position.

© 2023. Published by The Company of Biologists under the terms of the Creative Commons Attribution License (https://creativecommons.org/licenses/by/4.0/).

To demonstrate what happens at DL/L = 0.2, as requested by the referee, we repeated simulations with an increased gradient decay length of DL=50 micrometers; the length of the patterning domain remained unchanged at L=250 micrometers. The plots below show the resulting gradient variability analogous to Fig 1c,d in the original manuscript. For both gradient parameters, we still recover the identical scaling laws.

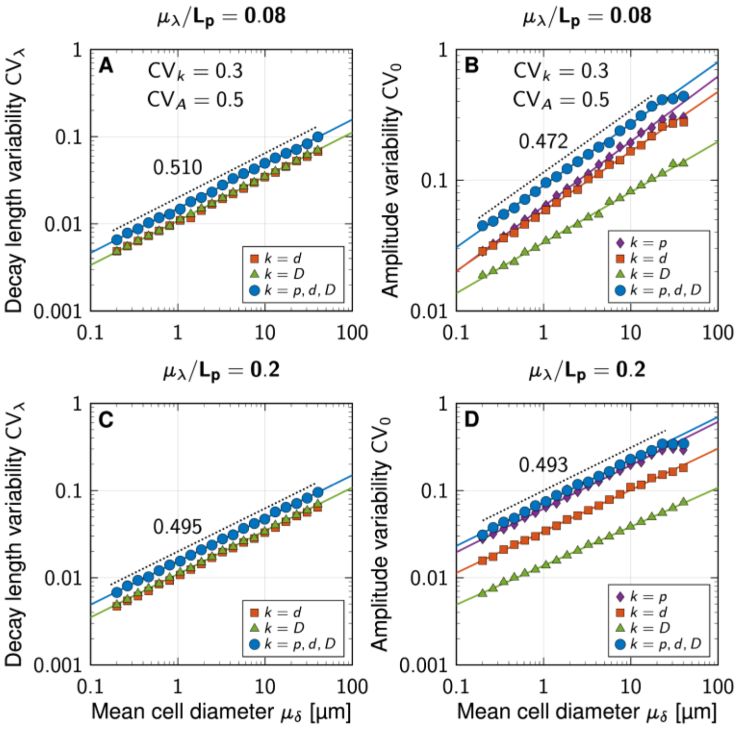

The source region is 25 microns in length and all cell diameters above 25 micron get defaulted back to 25 micron which explains the flatness lines in the region beyond mu_delta/mu_DL> 1

Thanks for pointing this out. We now mention this in the manuscript. Note that it’s the ratio mu_delta/L_s that matters, not mu_delta/mu_lambda. It just so happens in this case, that both are nearly equal, because L_s=5*mu_lambda/4 in our simulations.

Results:

Pg 2 (bottom left): In the git repository code, the morphogen gradients are fit to a hyperbolic cosines function (described in reference 19) which is not described in the main text. Having this in the main text would help readers understand why fig 1c has variation in d only, D only and all k parameters whereas fig 1d has variation with all individual parameters p, d and D and all k.

The reason why the impact of CV_p alone on CV_lambda is not plotted in Fig 1c is that it is minuscule. We now mention this in the figure legend. This follows from the fact that the gradient length lambda is determined in the patterning domain, whereas the production rate p sets the morphogen concentration in the source domain, and thus, the gradient amplitude, but not its characteristic length. This is unrelated to the functional form used to fit the shape of the gradients, be it exponential or a hyperbolic cosine. We mention that we fit hyperbolic cosines to the numerical gradients in section *Gradient parameter extraction* in the Methods section, and we refer the interested reader to the original reference [Vetter & Iber, 2022], which contains all mathematical details, should they be needed.

Figure 3b:

In figures where markers are overlapping perhaps the authors can use a "dot" to identify one set of simulations and a "o" to identify the ones under it. The way the plots are set up

© 2023. Published by The Company of Biologists under the terms of the Creative Commons Attribution License (https://creativecommons.org/licenses/by/4.0/).

currently makes it hard for the reader to understand where certain points on the plot are.

We use a color code to represent the readout strategy and different symbols to represent the cell diameter in Fig 3b. We agree that for the smallest of the cell diameters, the diamond-shaped data points lie so close that they are not easy to tell apart at first sight. For this reason, we chose different symbol sizes. We would like to keep the symbols as they are to maintain visual consistency with the other figures, which we think is an important feature of our presentation that facilitates the interpretation. Note that all our figures are vector graphics, which allow the reader to zoom in arbitrarily deep, and to easily distinguish the data points, as demonstrated in the closeup screenshot below. Note also that in this particular case, telling the data points apart is not necessary; recognizing that they are nearly identical is sufficient for the interpretation of our results.

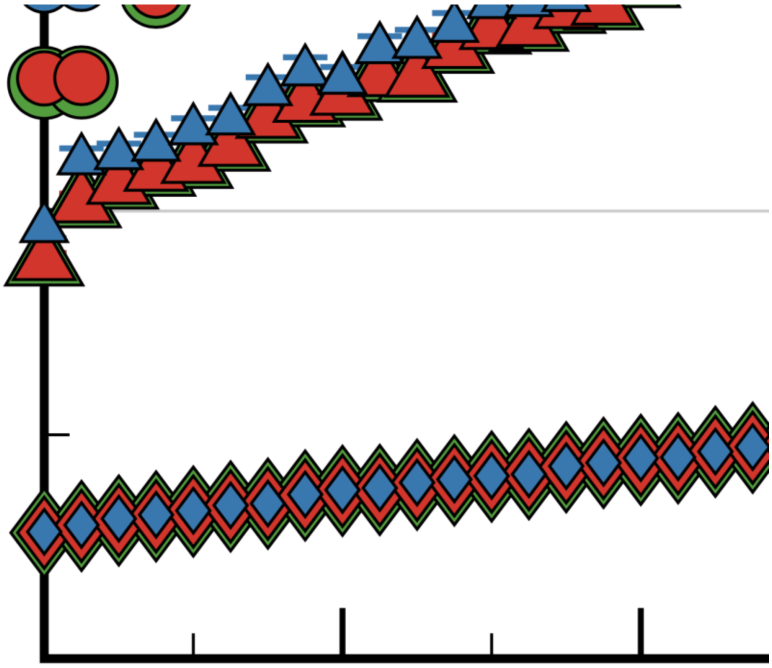

Methods:
The Methods can be more descriptive to include certain aspects of the simulations such as adjusted lambda which is only described in the code and not the main text or supplementary.

We apologize for this omitted detail. As shown in Fig. 8g in [Vetter & Iber, 2022], the mean fitted value of lambda drifts away from the prescribed value, depending on which of the kinetic parameters are varied, and by how much. To report the true observed mean gradient length in our results, we corrected for this drift in our implementation, as the referee correctly noticed. We now describe this in the methods section, and we have extended the methods also on other aspects.

Git code:
The git code function handles do not represent figure numbers and should be updated to make it easier for readers to find the right code

Thank you for pointing this out — it was an oversight from an earlier preprint version. The function names now correspond to the figure numbers.

Reviewer #1 (Significance (Required)):

This manuscript contributes certain key aspects to the patterning domain. The three most important contributions of this work to the current literature are: (1) the scaling relationships developed here are important, (2) the idea that PE increases at the tail-end of the morphogen profile is nicely shown and (3) Comparison of various readout

© 2023. Published by The Company of Biologists under the terms of the Creative Commons Attribution License (https://creativecommons.org/licenses/by/4.0/).

strategies.

Thank you for the positive assessment.

Reviewer #2 (Evidence, reproducibility and clarity (Required)):

Summary:

How morphogen gradients yield to precise patterning outputs is an important problem in developmental biology. In this manuscript, Adelmann et al. study the impact of cell size in the precision of morphogen gradients and use a theoretical framework to show that positional error is proportional to the square root of cell diameter, suggesting that the smaller the cells in a patterning field, the more precise patterns can be established against morphogen gradient variability. This result remains true even when cells average the morphogen signal across their surface or spatial correlations between cells are introduced. Thus, the authors suggest that epithelial tissues patterned by morphogen gradients buffer morphogen variability by reducing apical cell areas and support their hypothesis by examining several experimental examples of gradient-based vs. non-gradient-based patterning systems.

Major comments:

1. While the idea that smaller cells yield to more precise morphogen gradient outputs is attractive, it is unclear whether patterning systems use this strategy to make patterns more precise, as there are several mechanisms that could achieve precision. Do actual developmental systems use it as a mechanism to increase precision? Or precision is achieved through other mechanisms (for example, cell sorting as in the zebrafish neural tube; Xiong et al. Cell, 2013). Indeed, classical patterning work on Drosophila embryo suggest that segmentation patterns are of an absolute size rather by an absolute number of cells (Sullivan, Nature, 1987). According to the authors, the patterning stripes should be more precise when embryos have higher cell densities than in the wild-type, but stripes are remarkably precise in wild-type embryos. This is likely due to other precision-ensuring mechanisms (such as downstream transcriptional repressors, in this case).

We want to emphasize that our predictions concern the precision of the gradients, not the precision of their readout, which can be strongly affected by readout noise, as we will show in a forthcoming paper. Cell sorting can sharpen boundaries in the transition zone, but this would not address errors in target domain sizes and is thus different from gradient precision as we discuss it here. Also, cell sorting as observed in the zebrafish neural tube requires higher cell motility than what is observed in most epithelial tissues. The work by Sullivan, Nature, 1987, is concerned with patterning of the early *Drosophila* embryo, and the stripes are defined already before cellularisation. We are unfortunately not aware of any work that quantified gradient precision at different cell densities in epithelia. This would, of course, be highly interesting data and would indeed put our predictions to a test. We are, to the best of our knowledge, the first to propose this principle with the present work. We have now made these points and distinctions clearer in the revised manuscript. Thank you for bringing this up.

2. Their modeling approach is based on exponential gradients formed by diffusion and linear degradation, but in reality, actual morphogen gradients are affected by receptor and proteoglycan binding and are likely not simply exponential and/or interpreted at the steady state. Do the main results of the manuscript hold even for non-exponential gradients or before they reach a steady state?

We can confirm that our results also hold for non-exponential gradients, as they emerge for example when morphogen degradation is self-enhanced (i.e., non-linear). This result will be published in a follow-up study [BioRxiv: 10.1101/2022.11.04.514993], which we now cite in the concluding remarks in the revised manuscript.

The analysis of pre-steady-state gradients lies outside of the scope of the present work, and so the question as to whether our results are applicable to them as well, remains to be answered in

© 2023. Published by The Company of Biologists under the terms of the Creative Commons Attribution License (https://creativecommons.org/licenses/by/4.0/).

future research. We have added a comment on this to the discussion.

3. In their Discussion section, the authors note that several patterning systems, such as the Drosophila wing and eye discs, show smaller cells near the morphogen source relative to other regions in the tissue. This observation suggests a prediction of the authors' hypothesis that can be tested experimentally. In the Drosophila wing and eye discs genetic mosaics of ectopic morphogen sources (such as Dpp) can (and have) been made. Therefore, one could predict that the patterning outputs in a region of larger cross-sectional areas will be more imprecise than in the endogenous source. Since this is a theoretical paper, it is understandable that authors are not going to make this experiment themselves, but I wonder if they can use published data to test this prediction or at least mention it in the manuscript to offer the experimental biology reader an idea of how their hypothesis can be tested experimentally.

We appreciate that the referee would like to help us inspire the experimental community. Unfortunately, the problem with the proposal is that Dpp has been shown to result in a lengthening of the cells (and thus a smaller cell width) [Widmann & Dahman, J Cell Sci, 2009]. The Dpp gradient thus ensures a small cell width close to its source, which makes it virtually impossible to test this proposal experimentally in the suggested way. Nevertheless, we have added brief comments on potential experimental testing of our predictions to the discussion.

Other comments:

- The Methods section should be expanded and should include more details about how authors consider cell size in their simulations. As presented, I believe that experimental biologists will not be able to grasp how the analysis was done.

We have expanded on the technical details of our model in the methods section, in particular in relation to the cell size, as requested. To avoid being overly redundant with existing published descriptions of the modeling details [Vetter & Iber, 2022], we focus here on a description of what has not been covered already, and refer the interested reader to our previous publication. It is inevitable for any kind of work, be it theoretical or experimental, to be less accessible to experts in other disciplines, but we believe that the presentation of our results is independent enough of modeling aspects to be accessible to experimental biologists, too.

- Authors use adjectives such as 'little' as 'small' without a comparative reference. For example in the abstract, the authors say that apical areas "are indeed small in developmental tissues." What does "small" mean? This should be avoided throughout the text.

We thank the referee for raising this point. Where appropriate, we changed the phrasing accordingly to clarify what the comparative reference is. We leave all sentences unchanged where the statement holds in absolute terms. Note that in the substantially revised analysis on the impact of the different length scales involved in the patterning process, we now explicitly show with simulation data and theory that the absolute positional error increases with increasing absolute cell diameter.

Reviewer #2 (Significance (Required)):

Overall, I believe that the manuscript is well written and deserves consideration for publication. However, authors should consider the points outlined above in order to make their manuscript more accessible and relevant to the developmental biology community.

Thank you for the positive assessment.

Reviewer #3 (Evidence, reproducibility and clarity (Required)):

In their mansucript "Impact of cell size on morphogen gradient precision" the authors Adelmann, Vetter and Iber numerically analyse a one-dimensional PDE-based model of morphogen gradient

© 2023. Published by The Company of Biologists under the terms of the Creative Commons Attribution License (https://creativecommons.org/licenses/by/4.0/).

formation in tissues in which the cell sizes and cell-specific parameters locally affecting the gradient properties are varied according to predefined distributions. They find that the average cell size has the largest impact on the variance of the gradient shape and the read-out precision downstream, while other factors such as details of the readout mechanism have markedly less influence on these properties. In addition they demonstrate that averaging gradient concentrations over typical cell areas induces a shift of the readout position, which however appears to be insignificant (~1% of the cell diameter) for typical parameters.

Overall this manuscript is in very good shape already and tackles an interesting topic. I still would like the authors to address the comments below before I would recommend any publication. My main criticism pertains to some of the authors' derivations which, as I find, partly do deserve more detail, and to their conclusions about gradient readout precision.

Thank you for the positive assessment.

MAJOR COMMENTS

1 - p. 1, left column: The positional error of the readout position does not only depend on the variation of the gradient parameters, as suggested by the first part of the introduction. A very important factor is also the fluctuations due to random arrival of molecules to the promoters that perform the readout due to the limited (and typically low) molecule number. In fact, for positions very distant to the source of the gradient, this noise source is expected to be dominant over gradient shape fluctuations. Importantly, these fluctuations also arise for non-fluctuating, "perfect" gradient inputs if copy numbers of the morphogen molecules are limited (which they always are). This important contribution to the noise is neglected in the work of the authors. This is OK if the purpose is focusing on the origin and influence of the gradient shape fluctuations, but that focus should be clearly highlighted in the introduction, saying explicitly that noise due to diffusive arrival of transcription factors is not taken into account in the given work (see, e.g., Tkacik, Gregor, Bialek, PLoS ONE 3, 2008)

In the present work, only precision of the gradients, but not the readout itself is studied. We have now mentioned this more explicitly in the introduction. We also acknowledge the fact that the readout itself introduces additional noise into the system. We are currently finishing up work that addresses exactly this subject, which is outside of the scope of the present paper.

What may have led to misinterpretation of the scope of our work is that we called x_theta the readout position. x_theta defines the location where cells sense (i.e., read out) a certain concentration threshold, and is not meant to be interpreted as the location of a certain readout (a downstream transcription factor) of the morphogen. We have made this distinction clearer in the revised manuscript.

2 - p.1, right column: Why exactly are the parameters p, d, D assumed to follow a log-normal distribution? Such a distribution has been verified for cell size, but the rationale behind choosing it also for the named parameters should be explained, in particular for D. Why would D depend on local properties of the cell? Which diffusion / transport mechanism precisely is assumed here?

The motivations for the used log-normal distributions for the kinetic parameters are the following:
1) The morphogen production rates, degradation rates and diffusivities must be strictly positive. This rules out a normal distribution.
2) The probability density of near-zero kinetic parameters must vanish quickly, as otherwise no successful patterning can occur. For example, a tiny diffusion coefficient would not enable morphogen transport over biologically useful distances within useful timeframes. This rules out a normal distribution truncated at zero, because very low diffusivities would occur rather frequently for such a distribution.
3) Given the absence of reports on distributions for p, d, D from the literature, we chose a plausible probability distribution that fulfills the above two criteria and possesses just two parameters, such that they are fully defined by a mean value and coefficient of variation. This is given by a lognormal distribution.

© 2023. Published by The Company of Biologists under the terms of the Creative Commons Attribution License (https://creativecommons.org/licenses/by/4.0/).

Our results are largely independent of the exact choice of probability distribution assumed for the kinetic parameters, under the constraints mentioned above. To demonstrate this, we have repeated a set of simulations with a gamma distribution with equal mean and variance as used for the lognormal distribution. Below are some simulation results for a gamma distribution with shape parameters a = 1/CV^2 and inverse scale parameter b = mu*CV^2 with CV = 0.3 as used in the results shown in the paper. As can be appreciated from these plots, the results do not change substantially, and our conclusions still hold. As we believe this information is potentially relevant for the readership of our paper, we have added this result and discussion to the supplement and to the conclusion in the main text.

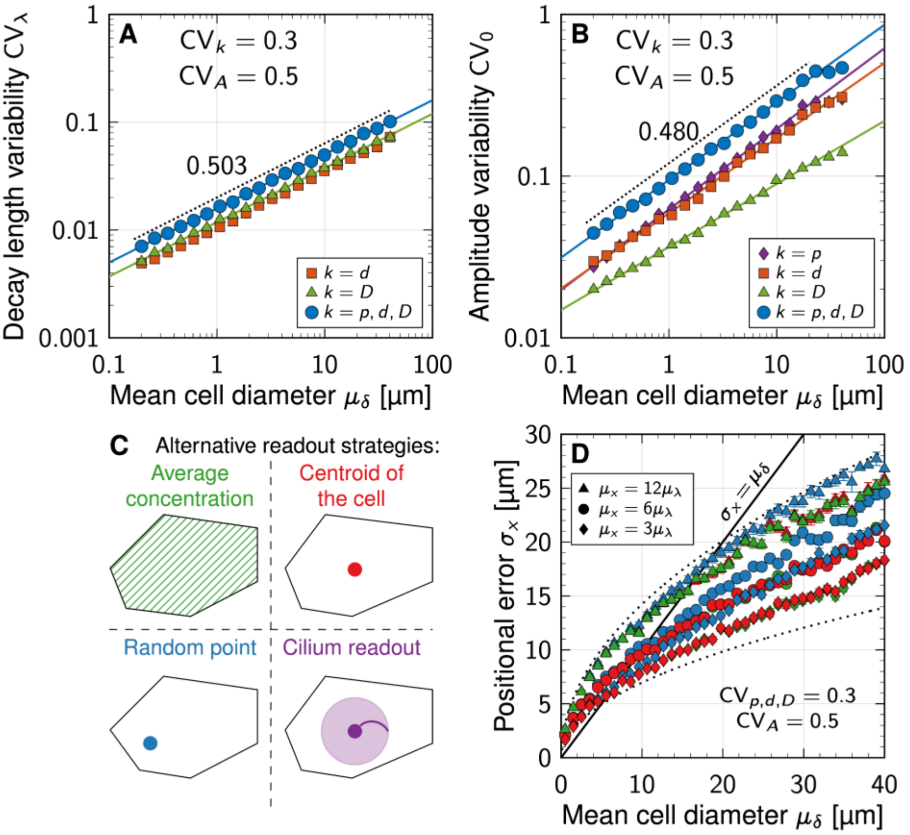

We assume extracellular, Fickean morphogen diffusion with effective diffusivity *D* along the epithelial cells, as specified by Eq. 2. We now state this more explicitly just below Eq. 2 in the revised manuscript. Cell-to-cell variability in the effective diffusivity may arise from effects that alter the effective diffusion path and dynamics along the surface of cells, which we do not model explicitly, but lump into the effective values of *D*. Such effects may include different diffusion paths (different tortuosities) or transient binding, among others.

Moreover, is there any relationship between A_i and p_i, d_i and D_i, or are these parameters varied completely independently? If yes, is there a justification for that?

The parameters are all varied independently, as written in the paragraph below Eq. 2 on the first page (“*drawn for each cell independently*”). To our knowledge there is no reported evidence for correlations between cell areas, morphogen production rates, degradation rates, or transport rates across epithelia, that we could base our model on. The choice of independent cell parameters therefore represents a plausible model of least assumptions made. Note that we explore the effect of potential spatial correlations in the kinetic parameters between neighboring cells in the section *“The effect of spatial correlation”*, finding that such correlations, if at all present, are unlikely to significantly alter our results.

3 - p. 2, right column, section on "Spatial averaging": First of all, how is "averaging" exactly defined here? Do the authors assume that the cells can perfectly integrate over their surface

© 2023. Published by The Company of Biologists under the terms of the Creative Commons Attribution License (https://creativecommons.org/licenses/by/4.0/).

in the dimensions perpendicular to their height? If yes, then this should be briefly mentioned here. Secondly, the shift \Delta x calculated by the authors ultimately seems to trace back to the fact that the cells average over an_exponential_ gradient, whose derivative also is exponential, such that levels further to the anterior from the cell center are higher (on average) than levels to the posterior of it. I suppose, therefore, that a similar calculation for linear gradients would not lead to any shift. If these things are true they deserve being mentioned in this part of the manuscript because they provide an intuitive explanation for the shift. Thirdly, in Fig. 2A the cell sizes seem exaggerated with respect to the gradient length. This seems fine for illustrative purposes, but if it is the case it should be mentioned. Also, I believe that this figure panel would benefit from showing another readout case where the average concentration e.g. in cell 1 maps to its corresponding readout position, in order to show that this process repeats in every cell. Moreover, it could be indicated that in the shown case C_\theta matches the average concentration in cell 2 at the indicated position.

Spatial averaging is defined as perfect integration along the spatial coordinate over a length of 2r (which can generally be equal to, or smaller than, or larger than one cell diameter) as detailed in the supplementary material. In simulations, we use the trapezoid method for numerical integration to get the average concentration a cell experiences along its surface area perpendicular to their height.

The reviewer is correct, that the shift is a consequence of averaging over an exponential gradient. The average of an exponential gradient is higher compared to the concentration at the centroid of the cell, thus the small shift. This is mentioned e.g. in the caption of Fig. S1, but also in the main text (“*spatial averaging of an exponential gradient results in a higher average concentration than centroid readout*”). We have now added this information also to the caption of Fig. 2. As pointed out correctly by the referee, linear gradients would not result in such a shift. A brief comment on this has been added to the revised manuscript.

We now mention that the cell size is exaggerated in comparison to the gradient decay length for illustration purposes in the schematic of Fig. 2a, as requested.

Unfortunately, we had a hard time following the reviewer’s final point. We show a specific readout threshold concentration, C_theta, in Fig. 2a. A cell determines its fate based on whether its sensed (possibly averaged) concentration is greater or smaller than C_theta. In the illustration, cells 1 and 2 sense a concentration greater than C_theta, and all further cells sense a concentration smaller than C_theta. Cell fate boundaries necessarily develop at cell boundaries (here; between cells 2 and 3, red). Additionally, the readout position for a continuous domain, where morphogen sensing can occur at an arbitrary point along the patterning axis, is shown (blue). This position can be different from the one restricted to cell borders. Thus, different readout positions in the patterning domain result from the two scenarios, which is what the schematic illustrates. Given that our illustration seems to go well with the other referees, we are unsure in what way it could be improved.

As for the significance of the magnitude of the shift for typical parameters as calculated by the authors: I believe that it could be said more explicitly and clearly that under biological conditions the calculated shift overall seems insignificant, as it amounts to a small fraction of the cell diameter.

We have made this more explicit in the text.

Finally, and most importantly: The term "spatial averaging" can have a different meaning in developmental biology than the one employed by the authors. While the authors mean by it that individual cells average the gradient concentration over their area, in other works "spatial averaging" typically means that individual cells sense "their" gradient value (by whatever mechanism) and then exchange molecules activated by it, which encode the read-out gradient value downstream, _between_ neighboring cells, in order to average out the gradient values "measured" under noisy conditions. The noise reduction effect of such spatial averaging can be very significant, as evidenced by this (incomplete) list of works which the authors can refer to:

- Erdmann, Howard, ten Wolde, PRL 103, 2009

© 2023. Published by The Company of Biologists under the terms of the Creative Commons Attribution License (https://creativecommons.org/licenses/by/4.0/).

- Sokolowski & Tkacik, PRE 91, 2015
- Ellison et al., PNAS 113, 2016
- Mugler, Levchenko, Nemenman, PNAS 113, 2016

The main point, however, is that this is a different mechanism as the one described by the authors, and this should be clearly mentioned in order to distinguished them. I would therefore also advise the authors to make the section title more precise here, by changing "Spatial averaging barely affects ..." to "Spatial averaging across the cell area barely affects ..." for clarity.

Most theory development has previously indeed been done with the syncitium of the early *Drosophila* embryo in mind. However, most patterning in development happens in epithelial (or mesenchymal) tissues, where spatial averaging via translated proteins is not as straightforward and natural as in a syncitium. In fact, a bucket transport of a produced protein from cell to cell would be difficult to arrange (as upon internalization, degradation would have to be prevented), be subject to much molecular noise, and be rather slow. Our paper is concerned with patterning in epithelia, which we have now stated more clearly in the manuscript.

Regarding the section title: Our analysis does not only cover spatial morphogen averaging over the cell area, but it also includes averaging radii below (in the theory) and far above (in the theory and in the new Fig. 4c, previously 3c) half a cell diameter. With cilia of sufficient length *r*, epithelial cells could potentially average over spatial regions extending further than their own cell area, without need for inter-cellular molecular exchange between neighboring cells. This is the kind of spatial averaging we explored here. Restricting the section title to the cell area only would therefore be misleading. However, we agree with the referee that the distinction between different meanings of “spatial averaging” is important, and we now emphasize our interpretation and the scope of our work more in the revised text.

4 - p. 3, Figure 3: It would be good to highlight the fact that the colours in panel A correspond to the bullet colors in the other panels also in the main text.

We now added this also in the main text.

As to the comparison of different readout strategies: How exactly were the different readout mechanisms compared on the mathematical side? More precisely: How was the readout by the whole area matched (in terms of fluxes) to the readout at a single point, be it in the center of the cell or a randomly chosen point? How was it ensured that the comparison is done at equal footing?

Our model considers that a cell can sense a single concentration even if it is exposed to a gradient of concentrations. Assuming the French flag model is correct, a cell must make a binary decision based on a sensed concentration in order to determine its fate. The different readout strategies are hypothetical and simplified mechanisms for how a cell could, in principle, detect a local morphogen signal. It is unclear to us what the referee is referring to when mentioning “matching in terms of fluxes”, as there are no fluxes involved in the modeled readout strategies. We make no assumption on the underlying biochemical mechanism that would allow cells to implement one of the strategies. The main goal of this analysis was to determine whether various different sensing strategies had a significant effect on the precision of morphogen gradients experienced by cells. To assure that we can compare the different mechanisms at equal footing, we simulated gradients and then calculated from each gradient the readout concentration in each cell and for each of the methods.

p. 3, right column: "... similar gradient variabilities, and thus readout precision": Linking to comment 1 above, this is strictly speaking only the case when the only source of fluctuations in the readout is the gradient fluctuations. I would therefore leave this statement out.

To avoid confusion, we have removed parts of the sentence. Thank you for pointing this out.

5 - p. 3, section on positional error (right column): In this part I had most troubles following the

© 2023. Published by The Company of Biologists under the terms of the Creative Commons Attribution License (https://creativecommons.org/licenses/by/4.0/).

thoughts of the authors.

First of all, the measure that the authors use for the positional error is sigma_x / mu_lambda, i.e. the standard deviation of the readout position relative to the gradient length. The question is whether this is the correct measure. It should be specified what the motivation for normalizing by mu_lambda is. In the end, one could argue, what the cells really do care about would be that the developmental process can assign cell fates with single cell precision, for which the other measure shown in Eq.
(6) is the representative one. Now in contrast to the former measure, the latter actually increases with decreasing cell diameter.

We thank the referee for raising this point, and acknowledge that we have not presented this aspect well enough. We have rewritten the entire section and the discussion about biological implications. Instead of normalizing with a constant mean gradient length in the formulas and figures, which has left room for misinterpretation, we now instead varied all relevant length scales in the patterning system, to determine the impact of each of them independently on the positional error. We now show that the positional error increases (to leading order) proportionally to the mean gradient length, the square root of the cell diameter, the square root of the location in the patterned tissue, and inversely proportional to the length of the source domain. We support these new aspects with new simulation data (Fig. 2E-2H, Fig. 3D-G, Fig. S5, Fig. S6). As the positional error is now reported in absolute terms, rather than relative to a particular length scale, the question of the relevant scale is addressed. We now show that the absolute positional error increases with increasing absolute cell diameter.

We believe that this extension provides additional important insight into what affects the patterning precision. We thank the referee very much for motivating us to expand our analysis.

Secondly, even when the former measure (sigma_x / mu_lambda) is employed, Fig. 3(D) shows that while it decreases with decreasing cell diameters, in the regime of small diameters the std. dev. of the readout position becomes larger than the average cell diameter, which actually would mean that cell fates cannot be assigned with single-cell precision. While the authors later report both quantities for specific gradients, it should be clarified beforehand which of the measures is the relevant one.

This has now been addressed by considering absolute length scales as discussed at length in our answer to the previous point.

Moreover, in the following derivations, mu_x is not properly introduced. What exactly is the definition of that quantity? Is it the mean readout position? If yes, it is not clear why exactly it would be interesting and relevant to the cell. This should be properly explained in a way that does not require the reader to look up further details in another publication.

The referee is correct in that mu_x is the mean readout position. We apologize for not being clear enough on this, and have now defined this in the introduction together with the definition of sigma_x.

At the end of this section the authors come back to the sigma_x / mu_delta measure again and indeed point out that it increases with decreasing mu_delta, which causes a bit of confusion because the initial part of the section only talks about the increase of the pos. error with mu_delta. Overall I find that this section should be rewritten more clearly. Right now it leaves the reader with the "take home message" that small cells are good because they lead to smaller pos. error, but when the--in my opinion--relevant measure (sigma_x/mu_delta) is employed the opposite is the case. This is confusing and unclear about the authors' intentions in that part.

See the answer above. The “take-home message” is now reformulated in absolute terms regarding the effect of cell diameter, rather than relative to a certain choice of reference scale. Our new analysis revealed a new relative ratio that determines the positional error, mu_lambda/L_s. We now discuss this relative measure also regarding its biological significance. Once again, we thank the referee for pointing us at this source of confusion, the

© 2023. Published by The Company of Biologists under the terms of the Creative Commons Attribution License (https://creativecommons.org/licenses/by/4.0/).

elimination of which allowed us to improve our analysis.

Finally, the authors could also supplement the numbers that they name for the FGF8 and SHH gradients by the known numbers for the Bcd gradient in Drosophila, which has been studied excessively and constitutes a paradigm of developmental biology. Here mu_delta ~= 6.5 um, while mu_lambda ~= 100 um, such that mu_delta/mu_lambda < 1/15, which defines yet another regime than the other two gradients. It would be interesting to compare their respective numbers altogether, and also discuss the ones for Drosophila in view of the fact that in experiments sigma_x ~= mu_delta for this species.

While we appreciate that most theoretical work has been done for syncytia, this paper is concerned with patterning of epithelia, which have different patterning constraints, as also explained in a reply further above. We now make the scope of our work clearer in the revised manuscript. But as the referee points out, the diameter of the nucleus relative to the gradient length is such that gradients can be expected to be sufficiently precise.

6 - p. 4, section on the effect of spatial correlation: Here the authors chose to order the kinetic parameters in ascending or descending order. Is there any biological motivation for that particular choice? Other types of correlations seem possible, e.g. imposing the rule that successive parameter values are sampled starting from the previous value, p_i+1 = o_i +- delta_i+1 where delta_i+1 are random numbers with a defined variance.

In the simulations we go from zero correlation (every cell has independent kinetic parameters) to maximal correlation (every cell has the same parameters, resulting effectively in a patterning domain that consists of a single effective “cell”), see Fig. S3. Biologically plausible correlations in between these extremes should retain the same kinetic variability levels (same CVs) which we took from the measured range reported in the literature. We accomplish this by ordering the parameters after independently sampling the parameters for each cell from probability distributions with the desired CV. The motivation for this approach is that this produces a type of maximal correlation that still reflects the measured biological cell-to-cell variability, to demonstrate in Fig. S3, that even such a maximal degree of spatial correlation does not qualitatively alter our results. The kind of correlation that the referee suggests introduces a spatial correlation length that lies in between the extremes that we simulated. Since even for maximal correlation using the ordering approach, we find our conclusions to still apply, we have no reason to expect that intermediate levels of correlation would behave any differently.

The idea brought forward by the referee effectively introduces a correlation length scale. We discuss this case in the paper, noting that the positional error will scale as σ ~ √, where N^x is the number of cells sharing the same kinetic parameters. A correlation length scale will be proportional to *N* and will therefore simply uniformly scale the positional error accordingly, but will likely not reveal any new insight beyond that.

Moreover, using the idea of the referee as an additional way to introduce correlation is difficult to realise in practice, as we need to recover the mean and variance of the kinetic parameters, while ensuring strict positivity for each of them. A simple random walk, as proposed, would not lend itself easily to achieve this without introducing a bias in the distribution, because negative values need to be prevented. As explained in a reply further above, an important feature of the kinetic parameters is that they are not too small to prevent the formation of a meaningful gradient, which is not straightforward to ensure with the proposed method.

We acknowledge that there are different types of correlations conceivable, but we expect these correlations to lie between the two extremes that we present in the paper, which show no qualitative difference in the results.

7 - p.5, Discussion: "..., but with nuclei much wider than the average cell diameter". To be honest, I could not completely imagine what is meant with this sentence.
Intuitively, it seems that the nuclei cannot be larger than the cells, but I suppose that some kind of special anisotropy is considered here? In any case, this should be made precise.

© 2023. Published by The Company of Biologists under the terms of the Creative Commons Attribution License (https://creativecommons.org/licenses/by/4.0/).

The main tissues that are patterned by gradients are epithelia. Our paper focuses on such tissues. It is a well-known feature of pseudostratified epithelia that nuclei are on average wider than the cell width averaged over the apical-basis axis. Nature solves this problem by stacking nuclei above each other along the apical-basal axis, resulting in a single-layered tissue that appears to be a multi-layered stratified tissue when only looking at nuclei. For a schematic illustration of this, see Fig. 1 in [DOI: 10.1016/j.gde.2022.101916]. An image search for “pseudostratified epithelia” on Google yields a plethora of microscopy images. Right at the end of the quote recited by the referee, we also cite our own study [Gomez et al, 2021], which quantifies this in Fig. 5.

Moreover, I find that the conclusion that morphogen gradients "provide precise positional information even far away from the morphogen source" goes to far based on the authors' work, precisely for the fact input fluctuations due to limited morphogen copy number, which can become detrimentally low far away from the source, are not considered, neither the timescales needed to both establish and sample such low concentrations far away from the source. While thus, according to the work of the authors, the fluctuations in the morphogen signal may be favorably small, these other factors are supposed to exert a strong limit on positional information. This conclusion therefore seems unjustified and should be toned down, or even better taken out and replaced by a more accurate one, which only focuses on the gradient shape fluctuations, not on the conveyed positional information.

There is no evidence so far that morphogen gradient concentrations become too low to be sensed by epithelial cells, to the best of our knowledge. What we show is that the gradient variability between embryos remains low enough that precise patterning remains possible. Whether the morphogen concentration remains high enough to be read out reliably by cells is a subject that requires future research. Genetic evidence from the mouse neural tube demonstrates that the SHH gradient is still sensed at a distance beyond 15 lambda (SHH signalling represses PAX7 expression at the dorsal end of the neural tube) [Dessaud et al., Nature, 2007], where an exponential concentration has dropped more than 3-million-fold.

As the referee correctly recites, we state that “morphogen gradients remain highly accurate over very long distances, providing precise positional information even far away from the morphogen source”. This statement is restricted to the positional information that the *gradients* convey, and does not touch potentially precision-enhancing or -deteriorating readout effects, nor does it concern the absolute number of morphogen molecules.

Positional information goes through several steps. The gradients themselves convey a first level of positional information, by being variable in patterning direction, as quantified by the positional error. This is what we draw our conclusion about. This positional information from the gradients can then be translated into positional information further downstream, by specific readout mechanisms, inter-cellular processes, temporal averaging, etc. About these further levels of positional information, we make no statement.

We therefore disagree that our conclusion is unjustified. In fact, we have phrased it exactly having the limited scope of our study in mind, making sure that we restrict the conclusion to the gradients themselves.

MINOR COMMENTS
- p. 1: "and find that positional accuracy is _the_ higher, the narrower the cells". (This sentence, however, should be anyhow revised in view of major comment 5 above.)

We have added “the”.

- p. 4: "... with an even slightly smaller prefactor."

We have removed “even”.

Reviewer #3 (Significance (Required)):

I believe that this work is significant to the community working on the theoretical

© 2023. Published by The Company of Biologists under the terms of the Creative Commons Attribution License (https://creativecommons.org/licenses/by/4.0/).

foundations of morphogen gradient precision in developmental systems. The main interesting findings are that small cell diameters lead to smaller positional error (although the relevant measure should be clarified according to my comment no. 5), and that the gradient shape fluctuations are surprisingly robust with respect to the readout mechanism.

Its limitations consist of the fact that the impact of small copy numbers on the readout and associated timescales are neglected, such that the findings of the authors on gradient robustness cannot be simply transferred by simple conversion formulas to readout robustness / positional information. Comment 5 goes hand in hand with this, as a different conclusion may emerge depending on how the relevant positional error measure is defined. This should be fixed by the authors as indicated in the main part of the report.

Thank you for your assessment.

Original submission

First decision letter

MS ID#: DEVELOP/2023/201702

MS TITLE: Impact of cell size on morphogen gradient precision

AUTHORS: Jan Andreas Adelmann, Roman Vetter, and Dagmar Iber

I have contacted two of the previous referees. The referees' comments are appended below, or you can access them online: please go to BenchPress and click on the 'Manuscripts with Decisions' queue in the Author Area.

The overall evaluation is positive and we would like to publish a revised manuscript in Development, provided that the referees' comments can be satisfactorily addressed. Please attend to all of the reviewers' comments in your revised manuscript and detail them in your point-by-point response. If you do not agree with any of their criticisms or suggestions explain clearly why this is so. If it would be helpful, you are welcome to contact us to discuss your revision in greater detail. Please send us a point-by-point response indicating your plans for addressing the refereeâ€™s comments, and we will look over this and provide further guidance.

Reviewer 1

Advance summary and potential significance to field

I have summarized the advance made in this paper in my original review for the paper in Review Commons.

Comments for the author

In this revised manuscript, the authors addressed my concerns with the first submission to Review Commons. I do have a few minor comments:

There is potentially a typo in Figure2F, in which it says CV0 is proportional to negative 1/Ls, but clearly CV0 and Ls are both positive parameters

In the wing disc section, more recent reviews could be cited:
- Tripathi, Bipin Kumar, and Kenneth D. Irvine. "The wing imaginal disc." Genetics 220.4 (2022): iyac020.

© 2023. Published by The Company of Biologists under the terms of the Creative Commons Attribution License (https://creativecommons.org/licenses/by/4.0/).

- Restrepo, Simon, Jeremiah J. Zartman, and Konrad Basler. "Coordination of patterning and growth by the morphogen DPP." Current Biology 24.6 (2014): R245-R255.

In the section describing the wing disc (High precision of scaled patterns…), the authors introduce many new ideas and names with numbers, parameters, and positions in the wing disc. To make the point more clear, it would be better to have a schematic of the wing disc that illustrates each of these features introduced. Fig. 5A is not adequate: omb and brk are not illustrated, 40-45% of La is not demarcated, 50% of Lp is not demarcated, presumptive positions of L2,L3,L5 are difficult to interpret. In general, Fig. 5A is too small. The colors are also difficult to read (dark yellow “sal” on light yellow background does not work well).

Reviewer 2

Advance summary and potential significance to field

The question of how morphogen gradients establish precise patterns of gene expression has been broadly discussed in developmental biology. Here, Adelmann et al. investigate the impact of cell size in the precision of morphogen gradients using a theoretical framework. They show that the positional error in the morphogen is proportional to the square root of cell diameter at the apical surface. The authors suggests that precision to fluctuating morphogen concentrations can be buffered by reducing apical cell size, even when cells average the morphogen signal across their surface or spatial correlations between cells are introduced. As a proof of principle, the authors support their hypothesis by examining experimental examples of gradient-based vs. non-gradient-based patterning systems. Finally, the authors use their findings to try to explain the emergence of pseudstratification, an interesting phenomenon in epithelial biology.

Comments for the author

This is the second time that I examine the manuscript and I believe that the the manuscript has been improved, and the new discussion on pseudostratification is a good addition to the paper. However, I still have two main comments and other minor comments that in my opinion will make the manuscript more readable, relevant, and clearly accessible to the average biology reader.

Main comments:

1. I still think that the main prediction of the paper can be compared to previously published data in which cell size, but not overall tissue size, has been modified genetically. For example, in Drosophila this has been done either in genetic mosaics or in whole compartments (see for example Neufeld, et al. Cell 1998). In this system, the predictions of the authors can be directly tested in the context of DPP patterning, for example. I am not suggesting the authors to analyze these data in this paper, but this is really relevant and should be further discussed in the paper. There may be experimental data already out there that may confirm or refute the authors' predictions.

2. Throughout the manuscript, the authors discuss their numerical findings with a specific biologically-relevant model system (e.g. the mouse neural tube as in page 3, first column, or in the last paragraph of the subsection "The effect of spatial correlation," related to pseudostratification in epithelia). Ideally, these interpretations of their results should be moved to the discussion section. However, an easier solution that I strongly suggest authors to follow is to merge the Results and Discussion sections into a single one and add a subtitle to the current Discussion section.

Other comments:

- In the abstract, the sentence “We numerically determine the impact of the cell diameter, gradient length, and the morphogen source on the variability of morphogen gradients and show that the positional error increases with the gradient length relative to the size of the morphogen source, and with the square root of the cell diameter and the readout position” is too long and confusing. Break into two sentences.

© 2023. Published by The Company of Biologists under the terms of the Creative Commons Attribution License (https://creativecommons.org/licenses/by/4.0/).

- In the introduction, the sentence “We analyse the impact of various length scales present in the epithelium, such as the cell diameter and source size, as well as spatial averaging, on morphogen gradient variability, finding that positional accuracy is higher, the narrower the cells and the larger the morphogen source” is again too long. Add a period after “variability” and then another sentence with the conclusion.

- In Fig. 2, colors need to be better explained in the legend. The authors seem to explain this saying “with kinetic variability only in the parameters indicated by different symbols” but this may not be clear. In addition, the authors should avoid saying in the legend what they see in the plots; they should describe this only in the Results section (e.g., F Except when only the diffusion coefficient varies (green), greater source lengths reduce morphogen amplitude variability). The numbers in A and B correspond to the fit of the blue curve, but this is not clear from the legend.

- In the beginning of the last paragraph of page 3, Colours in panel 4A correspond to the colours in panels 4B- G), should be removed as it is already clear from the legend of Fig. 4.

- Fig. 2 A and B show the main result of the paper, namely the impact that cell diameter has on decay length and amplitude variability. However, the range in which mean cell diameter is varied (from 0.1 to 100 μm!) is huge. I believe the authors want to include a broad range of values from different biological systems, but this is somehow misleading, because the purpose of the result is to say that cell size affects the morphogen’s decay length and amplitude variability. Still, I believe that the main result of the paper holds, but the authors should clarify this.

- In the last paragraph subsection "Gradient variability increases with cell size, but not with physiological levels of cell area variability" (page 3), the authors report some parameter values for the mouse neural tube, but it is unclear in which simulations they used these parameters.

First revision

Author response to reviewers' comments

Reviewer 1 Advance Summary and Potential Significance to Field:
I have summarized the advance made in this paper in my original review for the paper in Review Commons.

Reviewer 1 Comments for the Author:
In this revised manuscript, the authors addressed my concerns with the first submission to Review Commons. I do have a few minor comments:

There is potentially a typo in Figure2F, in which it says CV0 is proportional to negative 1/Ls, but clearly CV0 and Ls are both positive parameters

As detailed in Table S1, there is a positive offset b in the fit $CV_0 = a/L_s + b$. For the one curve shown in Fig. 2F for which we wrote the asymptotic scaling as $CV_0 \sim -1/L_s$, the prefactor a is negative. We acknowledge that this might be unnecessarily confusing for some of the readership, and we have removed this annotation from the figure. We retain the other annotation, $CV_0 \sim 1/L_s$, as it applies generally, with an omitted prefactor that can be positive or negative.

In the wing disc section, more recent reviews could be cited:

- Tripathi, Bipin Kumar, and Kenneth D. Irvine. "The wing imaginal disc." Genetics 220.4 (2022): iyac020.

- Restrepo, Simon, Jeremiah J. Zartman, and Konrad Basler. "Coordination of patterning and growth by the morphogen DPP." Current Biology 24.6 (2014): R245-R255.

© 2023. Published by The Company of Biologists under the terms of the Creative Commons Attribution License (https://creativecommons.org/licenses/by/4.0/).

Thank you. We now include these references in the revised manuscript.

In the section describing the wing disc (High precision of scaled patterns…) the authors introduce many new ideas and names with numbers, parameters, and positions in the wing disc. To make the point more clear, it would be better to have a schematic of the wing disc that illustrates each of these features introduced. Fig. 5A is not adequate: omb and brk are not illustrated, 40-45% of La is not demarcated, 50% of Lp is not demarcated, presumptive positions of L2,L3,L5 are difficult to interpret. In general, Fig. 5A is too small. The colors are also difficult to read (dark yellow "sal" on light yellow background does not work well).

Thank you for pointing this out. We have now adapted the figure and think that it is clearer now.

Reviewer 2 Advance Summary and Potential Significance to Field:
The question of how morphogen gradients establish precise patterns of gene expression has been broadly discussed in developmental biology. Here, Adelmann et al. investigate the impact of cell size in the precision of morphogen gradients using a theoretical framework. They show that the positional error in the morphogen is proportional to the square root of cell diameter at the apical surface. The authors suggest that precision to fluctuating morphogen concentrations can be buffered by reducing apical cell size, even when cells average the morphogen signal across their surface or spatial correlations between cells are introduced. As a proof of principle, the authors support their hypothesis by examining experimental examples of gradient-based vs. non-gradient-based patterning systems. Finally, the authors use their findings to try to explain the emergence of pseudstratification, an interesting phenomenon in epithelial biology.

Reviewer 2 Comments for the Author:
This is the second time that I examine the manuscript and I believe that the manuscript has been improved, and the new discussion on pseudostratification is a good addition to the paper. However, I still have two main comments and other minor comments that in my opinion will make the manuscript more readable, relevant, and clearly accessible to the average biology reader.

Main comments:

1. I still think that the main prediction of the paper can be compared to previously published data in which cell size, but not overall tissue size, has been modified genetically. For example, in Drosophila this has been done either in genetic mosaics or in whole compartments (see for example Neufeld, et al. Cell 1998). In this system, the predictions of the authors can be directly tested in the context of DPP patterning, for example. I am not suggesting the authors to analyze these data in this paper, but this is really relevant and should be further discussed in the paper. There may be experimental data already out there that may confirm or refute the authors' predictions.

Unfortunately, we have not been able to find any data that compares patterning precision in developmental systems with different cell sizes. To address the referee's comment, we now suggest several ways of how our prediction could be tested experimentally, including the method introduced by Neufeld et al. In addition, we mention the possibility of altering the cell diameter using a tissue stretching device, as previously used in the *Drosophila* wing disc (Duda et al. 2019, Dev. Cell).

2. Throughout the manuscript, the authors discuss their numerical findings with a specific biologically-relevant model system (e.g. the mouse neural tube as in page 3, first column, or in the last paragraph of the subsection "The effect of spatial correlation," related to pseudostratification in epithelia). Ideally, these interpretations of their results should be moved to the discussion section. However, an easier solution that I strongly suggest authors to follow is to merge the Results and Discussion sections into a single one and add a subtitle to the current Discussion section.

https://journals.biologists.com/dev/pages/manuscript-prep states that Results and Discussion sections should be kept separate for Research Articles. Unless the editor wants us to deviate from

© 2023. Published by The Company of Biologists under the terms of the Creative Commons Attribution License (https://creativecommons.org/licenses/by/4.0/).

these instructions, we wish to follow them by using the Discussion section to place our results into the broader context of the current literature, while explaining the results and their relevance pertinent to specific questions in tissue development in the Results section.

Other comments:

-In the abstract, the sentence "We numerically determine the impact of the cell diameter, gradient length, and the morphogen source on the variability of morphogen gradients and show that the positional error increases with the gradient length relative to the size of the morphogen source, and with the square root of the cell diameter and the readout position" is too long and confusing. Break into two sentences.

The sentence is now split into two.

-In the introduction, the sentence "We analyse the impact of various length scales present in the epithelium, such as the cell diameter and source size, as well as spatial averaging, on morphogen gradient variability, finding that positional accuracy is higher, the narrower the cells and the larger the morphogen source" is again too long. Add a period after "variability" and then another sentence with the conclusion.

Thank you for pointing this out, we changed the sentence accordingly.

-In Fig. 2, colors need to be better explained in the legend. The authors seem to explain this saying "with kinetic variability only in the parameters indicated by different symbols" but this may not be clear. In addition, the authors should avoid saying in the legend what they see in the plots; they should describe this only in the Results section (e.g., F Except when only the diffusion coefficient varies (green), greater source lengths reduce morphogen amplitude variability). The numbers in A and B correspond to the fit of the blue curve, but this is not clear from the legend.

The figure legend has been improved to provide clearer explanations for the labels. Additionally, the captions for Figs. 2, 4, and 6 have been rephrased. The numbers in panels A and B of Fig. 2 are now better explained.

-In the beginning of the last paragraph of page 3, Colours in panel 4A correspond to the colours in panels 4B- G), should be removed as it is already clear from the legend of Fig. 4.

We have removed the sentence.

-Fig. 2 A and B show the main result of the paper, namely the impact that cell diameter has on decay length and amplitude variability. However, the range in which mean cell diameter is varied (from 0.1 to 100 µm!) is huge. I believe the authors want to include a broad range of values from different biological systems, but this is somehow misleading, because the purpose of the result is to say that cell size affects the morphogen's decay length and amplitude variability. Still, I believe that the main result of the paper holds, but the authors should clarify this.

Evolutionarily speaking, the entire range of cell diameters would, in principle, be possible. Our simulations allow us to characterise the impact that such epithelial cell diameters would have on patterning precision, and provide a rationale for the observed cell diameters and the emergence of pseudostratification in epithelia. Moreover, this approach allowed us to uncover the square-root scaling. Had we limited our analysis to a substantially narrower range, perhaps with a particular tissue in mind, we would not have arrived at the scaling relationships, which are at the core of our findings. It is in fact a strength of our modeling approach, that it allows us to cover such a wide parameter range. We believe that it would have been a shortcoming of our work, had we not covered a range wide enough to robustly infer the functional dependency. Power laws, as found here, are a prime example of a functional relationship that absolutely require data over several orders of magnitude to be detected with statistical confidence.

To make this clearer to a potentially diverse readership, we have added an introductory

© 2023. Published by The Company of Biologists under the terms of the Creative Commons Attribution License (https://creativecommons.org/licenses/by/4.0/).

sentence explaining the rationale behind this:

“We performed simulations covering a wide range of cell sizes, potentially larger than is relevant for a specific type of tissue or organism, to reveal the functional dependency of gradient variability on the cell diameter with statistical confidence. An increase in the average cell diameter …”

-In the last paragraph subsection "Gradient variability increases with cell size, but not with physiological levels of cell area variability" (page 3), the authors report some parameter values for the mouse neural tube, but it is unclear in which simulations they used these parameters.

We used these parameters in all reported simulations, except those where specific parameters were varied, as shown in the plots. We now mention this in the paragraph.

Second decision letter

MS ID#: DEVELOP/2023/201702

MS TITLE: Impact of cell size on morphogen gradient precision

AUTHORS: Jan Andreas Adelmann, Roman Vetter, and Dagmar Iber
ARTICLE TYPE: Research Article

Thank you for sending your manuscript to Development through Review Commons

I am happy to tell you that your manuscript has been accepted for publication in Development, pending our standard ethics checks.

© 2023. Published by The Company of Biologists under the terms of the Creative Commons Attribution License (https://creativecommons.org/licenses/by/4.0/).
